# Background, design and conceptual model of the cluster randomized multiple-component workplace study: FRamed Intervention to Decrease Occupational Muscle pain - "FRIDOM"

**DOI:** 10.1186/s12889-016-3758-6

**Published:** 2016-10-24

**Authors:** Jeanette Reffstrup Christensen, Thomas Viskum Gjelstrup Bredahl, Jenny Hadrévi, Gisela Sjøgaard, Karen Søgaard

**Affiliations:** 1 Department of Sports Science and Clinical Biomechanics, University of Southern Denmark, Campusvej 55, 5230 Odense M, Denmark; 2 Department of Community Medicine and Rehabilitation, Sports Medicine, Umeå University, 901 87 Umeå, Sweden

**Keywords:** RCT, Worksite, Health promotion, Implementation, Maintained effect, Sickness presenteeism, Sickness absenteeism, Exercise, Diet, Cognitive behavioral training

## Abstract

**Background:**

Several RCT studies have aimed to reduce either musculoskeletal disorders, sickness presenteeism, sickness absenteeism or a combination of these among females with high physical work demands. These studies have provided evidence that workplace health promotion (WHP) interventions are effective, but long-term effects are still uncertain. These studies either lack to succeed in maintaining intervention effects or lack to document if effects are maintained past a one-year period. This paper describes the background, design and conceptual model of the FRIDOM (FRamed Intervention to Decrease Occupational Muscle pain) WHP program among health care workers. A job group characterized by having high physical work demands, musculoskeletal disorders, high sickness presenteeism - and absenteeism.

**Methods:**

FRIDOM aimed to reduce neck and shoulder pain. Secondary aims were to decrease sickness presenteeism, sickness absenteeism and lifestyle-diseases such as other musculoskeletal disorders as well as metabolic-, and cardiovascular disorders – and to maintain participation to regular physical exercise training, after a one year intervention period. The entire concept was tailored to a population of female health care workers. This was done through a multi-component intervention including 1) intelligent physical exercise training (IPET), dietary advice and weight loss (DAW) and cognitive behavioural training (CBT).

**Discussion:**

The FRIDOM program has the potential to provide evidence-based knowledge of the pain reducing effect of a multi component WHP among a female group of employees with a high prevalence of musculoskeletal disorders and in a long term perspective evaluate the effects on sickness presenteeism and absenteeism as well as risk of life-style diseases.

**Trial registration:**

NCT02843269, 06.27.2016 - retrospectively registered.

**Electronic supplementary material:**

The online version of this article (doi:10.1186/s12889-016-3758-6) contains supplementary material, which is available to authorized users.

## Background

The consequences of unhealthy lifestyle constitute a major public health challenge in Western countries [1–3]. Factors such as physical inactivity and poor diet, can lead to overweight or obesity and consequently musculoskeletal pain. It has large economic consequences for society, in general, and for workplaces and the individual worker, in particular, in terms of reduced productivity, long-term sickness absence and premature exit from the labor market [4–7]. Unhealthy lifestyle and long-term sickness absence are common in low education jobs such as health care workers, a predominantly female job group also characterized by low physical capacity in terms of low muscle strength and aerobic capacity, as well as high prevalence of musculoskeletal pain and overweight or obesity [8–11]. Health care work consists of many hours of walking and standing with a wide variety of physical activities such as cleaning and patient handling, often involving large physical demands and peak forces [9, 12]. Studies suggest, that the high frequency of lifestyle diseases including musculoskeletal pain and sickness absence among health care workers, is caused by the mismatch of heavy work tasks and a low aerobic fitness level, low muscle strength and excessive body weight [8, 11, 13–16].

In a physically heavy job, high body weight imposes a high biomechanical strain on joints and muscles and a large body frame may hinder optimal ergonomic working postures during patient handling. Thus, obesity is a focal risk factor closely associated with a large number of lifestyle diseases also including musculoskeletal pain [17, 18].

Changes in humoral factors such as an increase in triglycerides, low-density lipoprotein (LDL) and cholesterol relate to inactivity and obesity, and are well known as cardiovascular risk factors [19, 20]. However, also for musculoskeletal pain metabolic markers have for long been an issue of investigation. Recent studies have in myalgic muscle interstitial fluid presented a number of metabolites associated with muscle pain [21–24]. However, a possible relation between humoral factors and the pain related muscle metabolites linking obesity and musculoskeletal pain have not been studied.

In the last decades, workplace health promotion (WHP) programs have emerged to improve the personal health of employees and to reduce organizational health-related expenses [25, 26]. A sizeable amount of evidence has accumulated towards the workplace as a promising setting for health promotion since it offers an efficient structure to reach large groups of people and makes use of natural social networks [26–28]. However, participation is required to ensure a clinical effect in the intervention. Adherence to work place exercise training is challenging and often displays a large number of drop-outs [29, 30]. However delivering WHP in working hours, paid by the workplace, lodges a possibility to a high participation to the WHP This should be supported by important factors such as leadership, organizational issues and instructors [31, 32]. Moreover, monotonous content, lack of inspiration and transferability to leisure time physical activity seem to be a barrier toward adherence [31].

This is supported by behavioral theory as e.g. the Self-Determination Theory (SDT) explaining the importance of autonomous motives, feeling of competence, control and relatedness for motivation and consequently adherence to a specific behavior [33]. Furthermore, the Theory of Planned Behavior explicit the need for perceived behavioral control (PBC) to enhance the possibility of progressing from intention to behavior or from work place physical exercise to regular leisure time exercise training [34, 35].

Educational level and occupational class is inversely associated with poor physical capacity and overweight or obesity, particularly among women [36, 37]. Since education and gender often are important factors in the stratification into certain occupational sectors, WHP have an opportunity to reach a specific target population and engage individuals who may otherwise face obstacles in participation in health-related activities [36–39]. WHP for health care workers therefore seem to be a legitimate attempt to reduce the growing health inequality in society by targeting this high-risk group.

The present protocol describes a FRamed Intervention to Decrease Occupational Muscle pain (FRIDOM). The main aim was to reduce neck and shoulder pain, secondary aim was to decrease lifestyle-diseases such as other musculoskeletal, metabolic-, and cardiovascular disorders. FRIDOM aimed eventually to decrease sickness presenteeism and sickness absenteeism. This was done through a multi-component intervention including 1) intelligent physical exercise training (IPET) [40], dietary advice and weight loss (DAW) and cognitive behavioural training (CBT). In a long term perspective the aim was to maintain participation to regular physical exercise training, beyond the 1 year intervention period. The entire concept was tailored to a population of female health care workers.

## Methods/Design

The FRIDOM program was composed of four parts: 1) A planning phase according to intervention mapping principles [41], 2) a feasibility study to reveal issues of implementation and intervention content, 3) a randomized controlled trial (RCT), and 4) a maintenance period. Below these components are further detailed.

### Intervention mapping and time frame

**Fig. 1:**
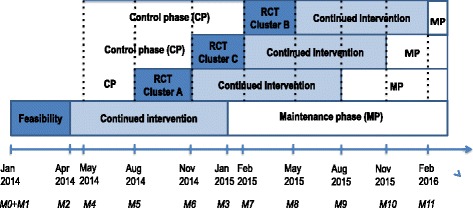
Time frame of feasibility and RCT study. *Legend:* The screening test on all 348 participants is numbered M0. The tests within the FRIDOM-feasibility study are numbered M1-M3 and the tests within the FRIDOM-RCT are numbered M4-M11

The main focus of the intervention was on sustainable behavioural life-style changes, therefore, a key point was a detailed planning of all phases to ease the final maintenance of the introduced health enhancing activities after the 12 months supervised intervention. The intervention planning included a need assessment in order to facilitate participation and another crucial part was to establish a firm organizational support for the intervention. Therefore all stakeholders in the municipality from the top level management to the individual health care worker were informed about the project and invited to information meetings on the intervention content, organization and management in the planning phase. The detailed planning was based on the following premisses: 1) The municipality should own the intervention and allow resources from the municipality to the intervention logistics. 2) The organization participated in a steering and working group and should allow time for meeting activities in these groups. 3) All employees were given the possibility to participate in the intervention at or nearby their geographical workplace during paid working time. 4) The intervention content should appeal to the participants. 5) The health effect of the intervention content should be evidence based. 6) The design should allow evaluation of the effect on primary and secondary outcomes, and 7) the intervention content should be integrated in the general work environment already taking place at the workplace [41]. The intervention mapping started around summer time 2013 and the subsequent planning resulted in commencing the feasibility study in January 2014 and the RCT study in August 2014, as shown in Fig. 1. The figure also presents duration of the different phases together with the time points for the measurements taken.

### The FRIDOM-Feasibility study

To prepare and facilitate the FRIDOM-RCT study a feasibility study was conducted which served several purposes: 1) To build confidence in the project internally in the municipality, 2) to allow a participatory approach, where experiences and suggestions from participants for improvement of the intervention content could be utilized in the final implementation in the RCT study, 3) to test if the intervention content planned for the RCT was practically suited for implementation in the municipality context, 4) to lean by and overcome organizational and practical barriers for participation and 5) evaluate both effectiveness and efficiency of the physical training program within the target group of health care workers.

Before the intervention in the feasibility study was initiated, all eligible employees were asked to fill out a screning questionnaire and participate in a baseline-screning-test. To allow for subsequent adjustment of the intervention content and mode of practical implementation, the feasibility study was timed approximal 7 months ahead of the RCT study, according to the same principal schedule as the RCT (Fig. 1). The maintenance period started January 2015 for the FRIDOM-Feasibility study (Fig. 1).

### The FRIDOM-RCT study

The FRIDOM-RCT was implemented as a single-blinded cluster randomized controlled trial (RCT) with a “stepped wedge” design (Fig. 1) [41, 42]. Content of the intervention had to be given repeatedly, and it was an important step to avoid changes in intervention delivery to the successive cohorts. In clinical intervention research, the RCT is considered the gold standard [43]. However, in WHP programs there appear to be three major obstacles: Firstly, the introduction of control clusters not receiving treatment may be considered unfair and can impair organizational commitment [42, 44]. Secondly, to maintain the intervention within all consenting employees, it was important that all employees were being offered the intervention at some point in time. Thirdly, from a workplace point of view, there could be a problem in implementing the program simultaneously to all consenting employees because of practical and logistical reasons [42, 44]. These three difficulties could all be overcome in the more feasible stepped-wedge design that enabled all consenting employees to receive the intervention.

### The FRIDOM-maintenance

**Fig. 2:**
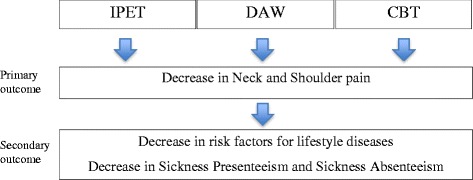
FRIDOM – Conceptual Model. *Legend:* IPET = Intelligent Physical Exercise Training, DAW = Dietary Advice and Weight loss, CBT = Cognitive Behavioral Therapy

The primary goal in the FRIDOM program was to reduce neck-shoulder pain (Fig. 2). Secondary goals were to decrease sickness presenteeism and sickness absenteeism by decreasing lifestyle-diseases through a multi-component intervention of intelligent physical exercise training (IPET) [40], dietary advice and weight loss (DAW) and cognitive behavioural training (CBT). Finally it was a long term goal to implement the intervention elements as permanent activities at the workplace, maintained beyond the 1-year intervention period. Therefore, the activities offered at the workplace should include not only baseline consenters, but also gradually involve new employees, employees returning to work from maternity leave, long-time sickness absence, sabbatical leaves as well as employees changing their mind regarding participation.

The maintenance of the activities introduced in the intervention was facilitated by a comprehensive reorganizational change in working plans in order to integrate a permanent one weekly work hour for intervention activities in the normal working plans for all teams of health care workers. After the 1-year intervention, the weekly planned training hour within work time was maintained, but employees were expected to work time off at another time point.

### Study population

#### Participants

The FRIDOM program was offered to the entire elderly care section in a medium size municipality in Jutland, Denmark. The health care section was divided in two main areas (RISOE and ROLI) with two different Area Managers. Each area was then divided into different centers for elderly people and retirement homes. These were spread out in five cities within the municipality. The cities are named Ebeltoft, Ryumgaard, Roende, Hornslet and Knebel. The main group of employees in the entire elderly care section was home care helpers (14 months of education) and home care assistants (21 months of education on top of the 14 months education as home care helpers) and in combination they are referred to as health care workers. All eligible personnel at the entire elderly care section (health care workers, nurses, activity staff, leaders, administrative staff and janitors) were invited to participate in the project to optimize motivation to comply with the intervention. Inclusion criteria were that participants: 1) were employed at the workplace for at least 15 h per week, 2) were permanently employed, or 3) had at least 12 months of work left before retirement. Exclusion criteria were long-term sick listed, pregnancy or working at a rehabilitation center, as these employees did not have work tasks within elderly care.

### Recruitment procedure

**Fig. 3:**
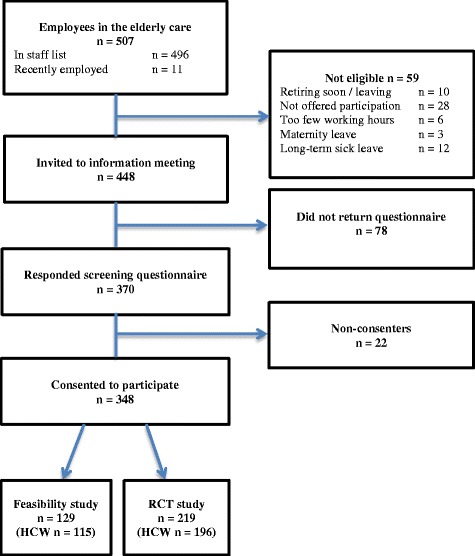
Flow chart of employees. *Legend:* HCW = Health Care Workers

Recruitment of eligible participants was based on the official list of staff that contained 496 employees (Fig. 3). Besides the list, the Area Managers were aware of 11 recently employed health care workers, and these were included as well, summarizing 507 employees on the elderly care section. The list was scrutinized excluding employees no longer employed, working less than 15 h a week, on either maternity or long-term sick leave. Additionally, 28 employees working in a separate rehabilitation center - all being either occupational- or physiotherapists – were not informed about the project and not offered participation. The remaining 448 employees were at the beginning of 2014 invited to information meetings during working hours where information about the FRIDOM program and a screening questionnaire were provided. A total of seven meetings were held to reach all eligible participants. Prior to the meetings, written information was distributed to the employees in a short information pamphlet. Managers supplied employees, who did not attend the information meeting with written information and a screening questionnaire. A total of 370 replied to the screening questionnaire and 348 consented to participate (Fig. 3). These were divided into 129 for the FRIDOM-Feasibility study and 219 for the FRIDOM-RCT study.

#### The FRIDOM-Feasibility study

The feasibility study involved 129 participants and was conducted among employees working in RISOE area. This part included both health care workers, working in centers for elderly people and retirement homes (together home care facilities) and employees working in the elderly’s private home in both day, evening and night shifts.

#### The FRIDOM-RCT

##### Randomization

The RCT clusters of 219 participants were individually allocated to a team by the first author JRC, according to the following criteria for minimizing variation within a team: 1) Geographical location of the workplace, 2) working at center/home care facilities or in the elderlies private home, and 3) day or evening/night shifts. This allocation resulted in 19 teams. To maximize motivation by social bonding with colleagues within a team, close relations with colleagues were taken into consideration. Subsequently, the 19 team were combined into three clusters of approximal same magnitude. In order to minimize contamination between clusters, priority was given to minimize variation in geographical location within a cluster. Additionally, the aim was to minimize variance of job exposure between clusters. Therefore balancing was perfomed between clusters regarding criteria 2 and 3 mentioned above, as well as ensuring a relatively even distribution of health care workers and nurses between the three clusters. This resulted in the three clusters composed as A: 5 teams from Roende and 2 teams from Ryumgaard, B: 5 teams from Hornslet, and C: 3 teams from Hornslet and 4 teams from Knebel. These tre clusters were randomized by author GS, using an opaque bag with the tree letters written on small paper cards. First letter drawn from the bag was A, then C, and finally B, which then defined the order of the clusters entering the stepped wedge design (Fig. 1).

##### Blinding

The outcome assessors were blinded to the employee’s cluster assignment. At follow-up testing, the employees were informed not to tell the outcome assessors what cluster they were assigned to. The outcome assessors were also trained to not discuss cluster allocation with the participants. All researchers and data analysts were blinded to cluster allocation. However, due to the content of the physical exercise training, participants and instructors could not be blinded to cluster allocation.

#### Intervention content

**Fig. 4:**
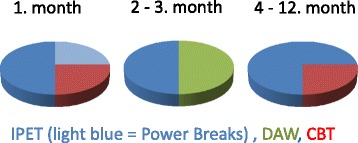
The multi-component intervention. *Legend:* IPET = Intelligent physical exercise training, DAW = Dietary advice and weight loss, CBT = Cognitive behavioural training. The pie-Chart displays the distribution of the one-hour weekly session

The 1-hour weekly multi-component intervention consisted of a combination of intelligent physical exercise training (IPET) including Power Breaks, dietary advice and weight loss (DAW) and cognitive behavioral therapy (CBT). An overview of the content over time is provided in Fig. 4.

#### Intelligent physical exercise training (IPET)

The principles for IPET were that the exercise training was adjusted to 1) occupational physical exposure, 2) physical capacity, 3) health status and 4) motivations and barriers. As FRIDOM targeted a homogeneous job group, the selection of exercises and training program was adjusted to the specific job demands of health care workers.

**Fig. 5:**
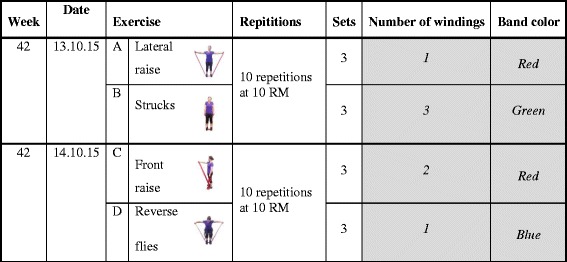
Examples of strengthening exercises performed as power breaks. *Legend:* RM = Repetitions Maximum

The physical exercise consisted of two elements: strength training exercises with elastic band as resistance (Power Breaks) and aerobic exercises. Four strength training exercises specifically targeting the neck and shoulder muscles were selected: Frontal raise, Lateral raise, Shrugs and Reverse flies (see Fig. 5). During the first month, 30 min of the one hour weekly session were devoted to familiarizing and correct performance of these exercises. This also included the choice of elastic band color and adjustment of length to provide the correct resistance of 12 RM and how to increase this to 10 RM. During months 2 and 3 the concept of a daily power break including 10 RM with 2 of the 4 exercises was introduced. The daily Power Breaks were now expected to be performed in addition to the weekly training sessions either during work time or at home. Aligned with the normalization process theory, participants were told to find an everyday suitable 5 min power break. Most optimal at the exact same time a day, in order to make the power breaks a new every day habit [45]. The aerobic exercises were inspired by the protocol developed in a previously workplace study [11]. The aerobic activities aimed at a level of 70 % aerobic capacity including warm up, circuit training and playing activities. In the first weekly sessions, aerobic activities were introduced in addition to the power breaks and with intensities around 50 % aerobic capacity. Over the first month the duration of the aerobic activities were gradually increased. In the second and third months, intensity in the aerobic exercises targeted a level of 70 % aerobic capacity to result in high calorie consumption to increase aerobic capacity and support weight loss. The last 9 months, focus was on being more physically active in leisure time – introducing outdoor power walks, hiking, running, biking or trying out different sports offered in local sport clubs. Complete lists of leisure time physical exercise possibilities in the municipality were presented to the participants. In all training clusters, a motivation was that the participants should decide and agree upon which aerobic exercise activities they would focus on. Four out of the 46 scheduled exercise sessions were replaced by visits to physical activity possibilities in the municipality. This was done to help the participants feel autonomous as highlighted by SDT [33] and to overcome the intention-behavior gap as explained by TPB [35] and to enhance motivation by introducing fun and playful elements to the IPET training.

#### Dietary advice and weight loss (DAW)

All participants were guided to ensure a healthy diet and prevent weight gain by adjusting courses according to the Danish Dietary recommendations, e.g. reduction of refined sugar, reduction of especially saturated fat, carbohydrates from primarily fiber-rich sources, and 600 g of fruit and vegetables per day. These guidelines were also consistent with new findings regarding weight loss [46]. Participants, who aimed to loose weight were encouraged to fill out a four days dietary record providing information on dietary preferences [47]. For each individual the resting metabolism was calculated, based on gender, age and weight and multiplied by a Physical Activity Level factor (PAL) of 1.8 to estimate the daily energy requirement [48]. ~1.000 cal were subtracted from the estimated daily energy requirements and the resulting value was used as individual calorie prescription. Individual guidance was provided for all meals with specific calorie amounts adjusted to suit the individual calorie prescription and dietary preferences. As shown in Fig. 4, the DAW-course was carried out during the second and third month, occupying approximately 30 min of the weekly session. From the fourth to the twelfth month, every session began with a weight check and individual dietary advices for those who still wanted to lose or maintain weight.

#### Cognitive behavioral training (CBT)

The CBT training was developed in a previous research project by Christensen et al. [11]. In short it was based on Linton’s model for coping with chronic pain modified to address discomfort during weight loss and to support a change to a more physically active lifestyle [49, 50]. The CBT was provided as group discussions and followed a specifically tailored guideline, containing exercises such as pro-and-con schemes and positive thinking strategies with homework between each session. During the first month, about 15 min of the weekly sessions were used on CBT (Fig. 4), helping the participants to make realistic weight loss targets and to support a change to a more physically active lifestyle. From the 4th to the 12th month of the intervention, about 15 min were spent on CBT in the weekly sessions. Focus was to reflect on dysfunctional attitudes and coping behaviors on how to continue healthy behaviors concerning both weight loss or weight maintenance and maintaining a physically active lifestyle. To support the aim of participant adherence to workplace physical exercise and regular leisure time physical exercise training, motivational initiatives based upon Self-Determination Theory (SDT) were included. To influence the feeling of autonomy, competence and control, the participants filled in a questionnaire expressing their wishes for leisure time physical exercise training after one month of intervention. Within the same questionnaire the participants were asked to describe possible barriers for their participation in leisure time physical exercise training but also possible solutions to overcome barriers. As a structured part of the RCT intervention and the cognitive training, the participants, discussed in groups on how to overcome barriers. These discussions were done to provide the participants the possibility to feel autonomous and competent and also to increase the relatedness to colleagues, Furthermore, motivational factors for their continued adherence were included in the discussion. During month two and three, the motives and barriers from the questionnaire concerning leisure time physical exercise were discussed again within the training clusters.

### Modifications from FRIDOM-Feasibility to the FRIDOM-RCT

Based on feedback from the participants a few changes were done before the intervention of the FRIDOM-RCT started. DAW and Power Breaks were introduced from the very start of the intervention. While the power breaks were to be performed in teams in the feasibility study, this was not feasible to integrate in the working days schedule. Instead, an individual dairy log with a detailed instruction was developed and handed out. Power Breaks could then be performed during workday or in leisure time, when the individual found it convenient. In the FRIDOM-Feasibility, it was an organisational challenge to arrange the weekly FRIDOM sessions at the workplace. Health care workers often change work days and work shifts, and the steering group decided that it was not realistic to constantly adjust work schedules to fit the FRIDOM intervention. Therefore, it was accepted that participation rate was lower due to vacation, days off or work pressure. Further a burn in period (~3 month) was added to the FRIDOM-RCT allowing for information and planning of the logistic to incorporate the intervention.

### Instructors

Due to Danish legislation, all employees were entitled to six weeks vacation. Thus, weekly training sessions were only carried out in 46 weeks and were all supervised with instructors. Most sessions took place at the workplace, but in 3–4 sessions, the training team visited local sports clubs in order to break barriers to leisure time exercise. The instructors were required to have a background as bachelors in sports, physiotherapy or public health. Every training team was supposed to be supervised with the same instructor during the entire year. During vacation or sickness in the instructor group, instructors would deputiz for each other, and no sessions were to be canselled. Instructors were paid from FRIDOM fundings in the intervention year.

### Outcome measures

Outcome measures were collected in four different categories: (Categorie 1) data drawn from workplace registrations, (categorie 2) questionnaires, (categorie 3) physiological tests and (categorie 4) adherence. Besides the physical tests, the physiological tests included blood samples of blood sucker, cholesterol and triglycerid. Categorie 1–4 tests were collected on all participants, in either the FRIDOM-feasibility or the FRIDOM-RCT study. At some timepoints, a full packages of each category were collected (Large test), while at other timepoints, only a subsets were collected (Mini test). Besides the Large tests and the Mini tests, a screning test was initially conducted. As a nested study, additional blod samples and objective heart and physical activity measures were collected on a smallere representative subsample.

With reference to Fig. 1, the outcome measures are specified for each test. The screening test is numbered M0. The FRIDOM-feasibility test measures are numbered M1-M3 and the FRIDOM-RCT test measures are numbered M4-M11.

In January 2014 (M0), all 348 participants participated in a screning test and filled out a screning questionnaire. Shortly after, the feasibility cluster (n = 129) was baseline-tested with a Large test in the categories 1–3 (M1). In April 2014 (M2), the feasibility cluster had a Large test at follow up, as they also had after one-years intervention, in January 2015 (M3).

In May 2014, the 219 RCT participants were baseline-tested with a Large test (M4) in the categories 1–3. In August 2014, the intervention of the first RCT-cluster commenced (M5), the second cluster in November 2014 (M6) and the third in February 2015 (M7) (Fig. 1). At each of these timepoints, and also in May 2015 (M8), the Mini test were performed on all three RCT-clusters and additional blod samples and objective heart and physical activity measures were collected on a smallere representative subsample only. The Mini tests, including objective heart and physical activity measures but without additional blod samples, continued within all three RCT-clusters every third months, in August 2015 (M9), November 2015 (M10) and February 2016 (M11). These tests were conducted as Mini tests after 3, 6 and 9 months intervention within each RCT-clusters, but as Large tests after 12 months intervention (Fig. 1).

### Workplace registrations (Category 1)

Information about age, sex, job seniority, weekly working hours, work shift (day shift, night/evening shift), care center or homecare, job type (home care helpers, home care assistants, nurses, leaders and administrative staff or other job categories) and educational level (unskilled, low skilled - <2 years of education - and high skilled - ≥2 years of education) were gathered from the workplaces own registration on their employees.

Information on sickness absenteeism was retrieved from a local database maintained by the executive director at the workplace. The records listed the beginning and the end dates of each sickness absence spell for each employee 12 months before intervention start and until the end of the 12 months intervention. Number of spells and days for each month were registered. The number of days with sickness absence represented workdays only. Maternity leaves and absences attributable to caring for a child were not included in the sickness absence registrations.

### Questionnaire (Category 2)

The FRIDOM program consisted of three different questionnaires. 1) A Large questionnaire given at baseline and followed-up after 1 year within each RCT-cluster, 2) a Mini questionnaire to be completed every third months, and finally 3) a Screening questionnaire distributed before randomization in January 2014 (Fig. 1). The Large questionnaire involved lifestyle behaviour and health (smoking, alcohol consumption, medicine use and sleeping behaviour), dietary intake (fruit, vegetables, fish, sugar and fat), physical activity during leisure time [51, 52], self-rated health and Stress-Energy Scale, SF-36 [53, 54], physical resources with five items: aerobic fitness, strength, endurance, flexibility and balance/coordination, readiness to change [55, 56], Cohens Perceived Stress Scale (PSS) [57], chronic diseases, Standardized Nordic Questionnaires of musculoskeletal symptoms last seven days and pain intensity on a numeric rank box scale [58], general self-efficacy [59], Tampa Scale of Kinesiophobia [60], Work ability Scale [61], perceived physical exertion [62], productivity by a single item in the World Health Organization Health and Work Performance Questionnaire (HPQ) [63] and Quantity and Quality of work [64].

The Mini questionnaire every third months consisted of eight questions, included the Standardized Nordic Questionnaires for the analysis of musculoskeletal symptoms last 7 days and pain intensity [58], physical capacity with five categories: aerobic fitness, strength, endurance, flexibility and balance/coordination [65] as well as readiness to change [55, 56] and general self-efficacy [59].

The Screening questionnaire consisted of a subset of items from the Large questionnaire. The purpose was to evaluating reach of the intervention within the RE-AIM framework [66], questions were selected to compare important factors related to family, health and pain status for consenters and non-concenters and responders and not-responders. Pain was measured by Standardized Nordic Questionnaires for the analysis of musculoskeletal symptoms last 12 months [58].

### Physiological tests (Category 3)

The FRIDOM program included three different test-batteries. A large test-battery measured at baseline and after one year, a Mini test every third months and a Screening test before randomization. All participants were tested with a Large test at baseline and after 1 year of intervention. For some of the tests with physical strain, some exclusion criteria were used: elevated blood pressure [67], angina pectoris, heart or lung prescription medication, current or previous serious illnesses and traumas, herniated disc, tennis elbow, golf elbow, carpal tunnel syndrome, significant level of musculoskeletal pain at the time of the test and pregnancy.

The large test lasted one hour and consisted of anthropometrical, health-related and physical capacity measures specified as the following. *Height* was measured to the nearest mm without shoes. *Body weight* was measured wearing light clothes, but without socks and shoes. One kilogram was subtracted from the weight measure to compensate for clothing. BMI was calculated as BMI = body weight/height^2^. References to BMI was according to the standardized classification of WHO: <25 kg/m^2^ = normal weight, 25–30 kg/m^2^ = overweight, ≥ 30 kg/m^2^ = obese [68]. *Body Fat* was measured using a bio impedance device (TANITA SC-330), which was set to 'standard' while body frame and the participant's age, height and gender were entered. *Waist circumference* was measured over the umbilicus standing up and with clothes on, using an ergonomic circumference measuring tape (Seco 203 Girth measuring tape) and clothes thickness was noted. *Blood pressure* iwas measured in seated position after 10 min of rest with an electronic blood pressure monitoring device (Artsana CS 410). Three measurements were performed one minute apart and an average calculated as in Appleyard M 1989 [67]. *Aerobic fitness* was measured with the Wattmax test using a Monark E327 bicycle ergometer and a pulse belt and was carried out according to Andersen LB 1995.

*Isometric maximal voluntary strength* was measured with a validated standardized setup [69], measuring maximal voluntary handgrip, shoulder elevation, back flexion and extension force [70]. The participants performed a minimum of three attempts with steady increasing force to reach maximum within 3–5 s. The test was repeated until a maximal of five contractions if the last attempt showed a more than 5 % increase. The participant rested at least 30 s between each attempt. The maximal attempt was recorded for further analysis. Standardized verbal command and encouragement was given to maximize the effort. *Handgrip* in both hands was measured using a grip strength measurer (Jamar) [71]. *Shoulder elevation* was measured with a Bofors dynamometer with the subject seated erect in a chair with legs hanging freely, arms hanging along the side and head facing forward. The distance from pressure point to sternoclavicular joint was measured as the moment arm [72]*. Back flexion and extension* were measured with the subject standing, facing/backing onto beam and support plate at the spina iliaca anterior superior. The Bofors dynamometer was fixed to pull horizontal with a belt positioned at the vertical level of m. deltoid insertion on the humerus. The distance from the belt to a line through the crista iliaca and lumbalcolumna (L4L5 level) was measured for the moment calculation [73]*. Balance test was performed as an* unilateral stance test with eyes open and participants were instructed to look directly ahead at a black spot placed approximately 2 m in front of them at eye height. The participants stood on the dominant foot (defined as the foot used for standing while kicking a ball) with the big toe of the non-dominant foot leaning against the medial malleolus of the dominant foot. The test was performed for 30 s [65]. Each participant was allowed three trials. If all three trials resulted in loss of balance before ending the test, the classification was recorded as failed. *Blood samples* were obtained from the participants as fasting mornings samples. One set of samples was for analysis of plasma using heparin prepared tubes, centrifuged and stored in room temperature until analyzed. Another set of serum samples were sampled for metabolomics analysis and handled in accordance with recommendations for accurate metabolite handling followed by gas chromatography mass spectrometry analysis [74]. Metabolomics analyses were only analyzed in a selected subsample before and after the first 3 months of the intervention in the RCT study, (i.e. M5 and M6 for cluster A, M6 and M7 for cluster C, and M7 and M8 for cluster B (Fig. 1)).

These were stored on ice water for 5–10 min, followed by 5 min of centrifugation at temperature-controlled conditions. Serum was pipetted and stored in three tubes and put on dry ice before transportation and finally stored in −80°. The plasma samples were analyzed for standard clinical measures of metabolites: high-density lipoprotein (HDL), low-density lipoprotein (LDL), total cholesterol, and glucose at accredited hospital laboratories.

**The Mini tests** lasted 15 min and included: blood pressure, body weight, BMI, body fat, waist circumference, and balance test.

**The Screening test** lasted 3 min and included information’s on weight, BMI and body fat collected with the same procedures as described within the large test.

### Adherence (Category 4)

The instructors registered participation in the 46 weekly multi-component training offered at the workplace. Performance of daily five minutes exercise with elastic bands (Power Breaks), color of the Thera band, and number of windings [1–3] were reported in a training diary (Fig. 5). The training diaries were collected every third months, and handed out so the dates in the diary were consistent with the next three months.

### Additional measures in a nested subsample

#### Heart and physical activity

Diurnal measurements consisting of physical activity and electrocardiogram were performed on a random subset of participants at M5, M6, M7 and M8 (Fig. 1). At each timepoint measurements included approx. 25 participants starting the active intervention and approx. 25 participants in waiting clusters or having finalized the intervention. Actigraph GT3X+ accelerometers (www.actigraphcorp.com) were used to quantify physical activity and was worn for 4 days. The monitor determined accelerations in three directions with a frequency of 30 Hz. Accelerations were sampled with a precision of 12 bit, with a dynamic range of ±6 G and stored as raw data. The accelerometer was mounted on the right thigh midway on the line between the iliac crest and the top of patella and orientated with the x-axis pointing downwards, y-axis horizontally to the left and z-axis horizontally forward. To insure firm position on the skin we used 3 M, Hair-Set, double sided adhesive tape and Fixomull, BSN medical. The Actigraphs were then covered with waterproof folio, initialized for recording and data were before analysis downloaded using commercial software (Actilife version 5.5). Physical activity was subsequently quantified as time in sitting, standing and walking using custom build software (Acti4).

Electrocardiogram for estimation of heart rate and heart rate variability was recorded using e-Patch data loggers (AMORS, AMS 3000 Delta Technology) initialised with gender and age using the commercial software Hasimed. The logger was mounted in a disposable adhesive pre-gelled electrodes patch, placed on sternum, 1 cm below clavicula. Recordings with a sensitivity of 0.25 mV covering 24 h of EKG stored with a frequency of 2000 Hz. Data were downloaded using the commercial software Hasimed and the off-line detection of the timing of the R-peaks in the QRS complex allowed for calculation of heart rate and heart rate variability.

During the diurnal measurements, participants were asked to follow their normal every-day schedule life and to note in a paper diary, working hours, sleeping and waking time, and if any of the monitors were removed before scheduled.

### Statistics

Analyses of outcome were performed both on the entire population and on the health care target population. The primary outcome measure was questionnaire based self-reported duration and intensity of neck pain with all other outcome measures considered secondary outcomes. For the Feasibility study the pre-post test were analyzed using paired t-test and repeated measures. For the RCT differences between the three clusters at baseline were to be tested with Pearson’s x^2^ for distribution in sex, education (health care workers), current smoking status and the dichotomized parameter for musculoskeletal symptoms in neck, shoulders, upper- and lower back. All other parameters were to be tested with a Student’s t-test. Changes over the 3 months intervention periods were compared with the changes over the corresponding 3 months control periods in an ANCOVA analysis in accordance to the intention-to-treat principle, i.e. all randomized participants were included in the analyses with missing values substituted with carried forward or backwards measured variables. Clusters, age and the investigated value at baseline were included as covariates. All results were given as mean (SD). *P* < 0.05 were considered statistically significant. SPSS statistics 23 was used for the statistical analysis.

In order to account for the combined effect within and between subjects and repeated measurements on the same subject, linear mixed effects models were used. Mixed effects models account for the inter-correlation of repeated measures. In this study, treatment as a categorical variable with two levels comparing intervention measurements with control measurements was included in the models as a fixed effect. Inter-correlation of repeated measures was included as a random effect. The analyses were further adjusted for baseline values of the respective outcome in a second model. Additionally, we used likelihood ratio tests to compare the models with and without a covariance structure. Analyses were based on the intention to treat principle including all eligible participants without imputations since mixed effects models inherently account for missing values.

Sample size for the RCT part was estimated using the method described by Woertman & colleagues for sample size calculation [75]. The study was powered to detect a between-groups mean difference in the primary endpoint of 1 point in neck and shoulder pain intensity, which has been considered a relevant change in the workplace context in terms of risk of sickness absence. The variance was set to 2.0 based on results from a Danish study [16] within a similar population, α to 0.05, power to 0.8, and an intra-cluster correlation coefficient to 0.05 in the three steps. We calculated that we needed 82 participants in total and with a dropout rate of up to 50 % we planned to recruit at least 160. However, to support implementation and support the workplace ownership the study design aimed to include all volunteer employees. This approach would improve generalization of the study findings to municipalities who would introduce similar health enhancing initiatives for their employees.

## Discussion

The FRIDOM program was designed to provide evidence-based knowledge of the pain reducing effect of a multi component WHP among a female group of employees with a high prevalence of musculoskeletal disorders. Musculoskeletal pain as well as overweight may be barriers for engaging in physical activity that can prevent life style diseases. Therefore the present study in a short-term perspective focus on physical activity to reduce neck shoulder pain and to support weight loss.

In a long term perspective health care workers with high physical demands present with a high risk for physical deterioration seen as low physical fitness, poor general health status, a high degree of sickness presenteeism and sickness absenteeism. Therefore the present study is designed to facilitate the implementation of the physical activities into normal daily life routines beyond the one-year intervention period.

Based on the IPET concept, we have in former studies successfully improved health among employees in a number of different job categories ranging from physically inactive to low, moderate, and finally, heavy physical work [40, 76]. The FRIDOM program specifically build on the former IPET results among health care workers adding a specific focus on influential factors for adherence to work place exercise training such as supporting organizational changes and facilitating an integrated gradual transfer from work place physical exercise to regular leisure time exercise training.

Other strengths of the FRIDOM program is the pragmatic design using a stepwise inclusion of all employers allowing for a strict effect evaluation in a 12 months perspective but still supporting a high degree of implementation at the workplace allowing for a long term effect. Another strong feature is the large feasibility study that as part of the intervention mapping allows a participatory approach to optimize integration of the intervention activities into the daily work routines and increase the sustainability beyond the intervention period.

All together the specific features of the FRIDOM program will provide evidence-based knowledge of the pain reducing effect of a multi component WHP among health care workers and in a long term perspective affect sickness presenteeism and absenteeism as well as risk of life-style diseases.

## Additional file

Additional file 1: SPIRIT 2013 Checklist. (DOC 437 kb)

## Abbreviations

CBT: Cognitive behavioral training
DAW: Dietary advice and weight loss
FRIDOM: FRamed Intervention to Decrease Occupational Muscle pain
HCW: Health care workers
HDL: High density lipoprotein cholesterol
IPET: Intelligent physical exercise training
LDL: Low density lipoprotein cholesterol
RCT: Randomized controlled trial
WHP: Workplace health promotion
